# Peering below the diffraction limit: robust and specific sorting of viruses with flow cytometry

**DOI:** 10.1186/s12985-016-0655-7

**Published:** 2016-12-01

**Authors:** Shea T. Lance, David J. Sukovich, Kenneth M. Stedman, Adam R. Abate

**Affiliations:** 1 Bioengineering and Therapeutic Sciences, University of California San Francisco, San Francisco, California USA; 2 California Institute for Quantitative Biosciences, University of California San Francisco, San Francisco, California USA; 3 UC Berkeley-UCSF Graduate Program in Bioengineering, University of California San Francisco, San Francisco, CA USA; 4 Center for Life in Extreme Environments, Biology Department, Portland State University, Portland, Oregon USA

**Keywords:** Droplet microfluidics, Next generation sequencing, Single virus genomics

## Abstract

**Background:**

Viruses are incredibly diverse organisms and impact all forms of life on Earth; however, individual virions are challenging to study due to their small size and mass, precluding almost all direct imaging or molecular analysis. Moreover, like microbes, the overwhelming majority of viruses cannot be cultured, impeding isolation, replication, and study of interesting new species. Here, we introduce PCR-activated virus sorting, a method to isolate specific viruses from a heterogeneous population. Specific sorting opens new avenues in the study of uncultivable viruses, including recovering the full genomes of viruses based on genetic fragments in metagenomes, or identifying the hosts of viruses.

**Methods:**

PAVS enables specific sorting of viruses with flow cytometry. A sample containing a virus population is processed through a microfluidic device to encapsulate it into droplets, such that the droplets contain different viruses from the sample. TaqMan PCR reagents are also included targeting specific virus species such that, upon thermal cycling, droplets containing the species become fluorescent. The target viruses are then recovered via droplet sorting. The recovered virus genomes can then be analyzed with qPCR and next generation sequencing.

**Results and Conclusions:**

We describe the PAVS workflow and demonstrate its specificity for identifying target viruses in a heterogeneous population. In addition, we demonstrate recovery of the target viruses via droplet sorting and analysis of their nucleic acids with qPCR.

**Electronic supplementary material:**

The online version of this article (doi:10.1186/s12985-016-0655-7) contains supplementary material, which is available to authorized users.

## Introduction

Viruses impact every form of life on earth, from applying evolutionary stresses to enhancing the transfer of genes between organisms [1–4]. Many human diseases are caused by viruses, including acute diseases like Ebola [5] and influenza [6], and chronic diseases caused by Epstein-Barr Virus (EBV) [7], Human Immunodeficiency Virus (HIV) [8], and Zika virus [9]. Studying viruses is thus important to human health, but also for elucidating the incredible mechanisms they’ve evolved to survive, replicate, and spread; these discoveries may lead to new molecular techniques and methods for treating disease. Studying viruses, however, can be challenging. They are usually much smaller than the diffraction limit of light and thus not directly visible with optical microscopy. They contain miniscule amounts of nucleic acid and protein, making direct sequencing or proteomic characterization of individual virus particles challenging [10]. To overcome these issues, the standard strategy is to culture the virus of interest to produce sufficient quantities for biological assays, such as gel electrophoresis, infection assays, or visualization with super-resolution or electron microscopy. However, like microbes, most viruses cannot be cultured [11], as this requires knowledge of which host cells the virus replicates in which, for most viruses, are also likely uncultivable [12, 13].

When a virus cannot be cultured, molecular methods are valuable. For example, viruses can be purified from a sample using filtration, flocculation, or density-dependent centrifugation, to recover particles of the appropriate size range, and the nucleic acids purified for PCR or next generation sequencing [14, 15]. This can be applied directly to environmental viruses and provides a genetic snapshot of organisms in that environment, and has yielded numerous insights into virus phylogeny and fundamental biology [14, 15]. However, viruses are also the most diverse organisms on the planet and viral samples often comprise sequences from trillions of entities, exceeding by orders of magnitude the limits of modern sequencers to sequence them [16]. As a result, such “shotgun” sequencing provides a sparse sampling of the system recovered as billions of short, hundred-base reads [17]. To extract meaningful biological insight from this complex data, the reads must be pieced into viral genomes, introducing substantial bioinformatic challenges that, often, cannot be overcome [10, 18, 19]. Most often, only genomic sequences for the most abundant organisms can be completely recovered and relatively little is learned about the vast number of new viruses present at low-to-moderate levels [20, 21]. To enhance the investigation of viral ecosystems, a method for culture-free purification of specific species would be valuable; however, as of yet, no method exists for specific sorting of viruses.

In this paper, we present specific and high throughput sorting of viruses, PCR-Activated Virus Sorting (PAVS). Using microfluidics, we encapsulate single particles from a population of diverse viruses into monodisperse double emulsion droplets. PCR reagents targeting specific genetic loci are also included, interrogating every droplet for these sequences. If a particle contains them, PCR signals are generated that cause the droplet to become fluorescent, making it sortable by double emulsion flow cytometry [22]. The recovered droplets can be ruptured and the material subjected to additional analyses, such as quantitative PCR or digital PCR and sequencing. The approach is simpler than antibody-based labeling and sorting of cells [23] because designing PCR TaqMan assays for specific detection of sequences is much easier than generating high affinity antibodies with which to label and sort single virus particles by flow cytometry. Moreover, the implementation of TaqMan PCR allows multiplexing to interrogate each virus for distinct sequences. Additionally, the use of TaqMan assays can be designed to incorporate “degenerate” sequences [24], allowing for the identification and recovery of diverse viral genomes. As we show, multiplexing can be used to measure the length distributions of viral genomes in a sample and is extendable to sequences that are not physically connected, such as genomic segments of viruses like influenza, or the 16S ribosomal RNA (rRNA) sequence of a bacterial cell harboring the target virus. Flow cytometric sorting has become a universal tool in cell biology and microbiology and PAVS allows it to be applied to viruses for the first time.

## Methods

### Preparation of viral samples

Bacteriophage T4 (T4) and bacteriophage ФX174 (ФX174) were obtained from Carolina Biological Supply. T4 is propagated by infection of *Escherichia coli* (*E.coli*) B (ATCC 11303) and ФX174 by infection of *E.coli* C (ATCC 13706). Bacteriophage lambda cI857ts is obtained from the lambda lysogen *E.coli* strain KL470 and propagated by infection of *E.coli* C600. Viral particles are collected from the supernate of the cultures and stored at 4 °C until experimentation. Initial viral stock concentrations are: T4 1×10^12^ pfu/mL, ФX174 1×10^10^ pfu/mL, Lambda 5×10^9^ pfu/mL.

### Microfabrication of devices

The microfluidic devices are fabricated using soft lithography in poly(dimethylsiloxane) (PDMS) [25]. SU-8 masters are fabricated by photolithography and used to mold PDMS devices by mixing PDMS polymer and cross-linker at a ratio of 11:1, pouring over the master, degassing to remove air bubbles, and baking at 75^o^C for 4 h to solidify. The device is extracted from the master with a scalpel, and inlet and outlet ports added with a 0.75 mm biopsy punch (Harris, Unicore). The device is washed with isopropyl alcohol and patted with Scotch® tape to remove debris prior to plasma bonding. The flow focus drop maker is bonded to a glass slide, baked at 75^o^C for 15 min, and treated with Aquapel to render the channels hydrophobic for water-in-oil emulsification. The double emulsion device is bonded and baked at 75^o^C for 48 h to completely revert the wettability to its native hydrophobic state. To pattern the channel wettability for double emulsification, select ports are blocked with Scotch tape, leaving others open for oxygen plasma treatment of 1 min [26].

### Encapsulation, PCR, and identification of viruses in single emulsions

To make single emulsions for viral detection, quantification, and genome length determination, the samples are first diluted in phosphate buffered saline and mixed with PCR reagents (Platinum Multiplex PCR Master Mix, Thermo Fisher) and PCR primers and TaqMan probes (Integrated DNA Technologies; IDT) specific for the species of interest. For detection or quantification, T4 viral particles are first diluted from the stock sample to ranges of 1×10^6^-5×10^8^ virus per sample prior to mixing with PCR reagent. For ApaI restriction enzyme digestion (NEB) or Fragmentase digest (New England Biolabs; NEB), Lambda DNA is treated enzymatically prior to the mixing with the PCR reagents. The oil phase of the emulsion consists of HFE-7500 fluorinated oil (3M) with 2% (w/w) PEG-PFPE amphiphilic block copolymer surfactant [27]. These solutions are loaded into syringes (BD 1 mL luer lock; 27G ½” needle), and the virus and PCR solution into a syringe atop 200 μL HFE-7500 oil; the oil acts as a hydraulic to push the solution into the device to accommodate for dead volumes, allowing nearly all of the solution to be used. The syringes are mounted onto pumps (New Era) with needles (BD), polyethylene tubing (PE-2) is affixed to the needles, and the syringes are primed by flowing at 5,000 μL/h prior to connecting them to the device. Flow rates are controlled with a custom Python script and set to 300 μL/h for the virus sample and 700 μL/h for the oil. The single emulsion droplets exit the device through PE-2 tubing and are collected into a 1.5 mL microcentrifuge tube. Single emulsions produced by this method are ~20 μm in diameter and monodispersed.

To prepare the single emulsion droplets for thermal cycling, the sample is transferred from the 1.5 mL microcentrifuge tube into 0.2 mL PCR tubes. The HFE-7500 oil is removed using a needle and is replaced with FC40-fluorinated oil with 5% Polyethylene glycol-perfluoropolyether (PEG-PFPE) amphiphilic block copolymer surfactant. The sample is cycled on a T100 thermal cycler (Bio-Rad) according to the Platinum Multiplex Master Mix instructions. After thermocycling, subsamples of single emulsions are subjected to visualization using a 6D High Throughput microscope (Nikon) with a 10× objective. Bright field, Cyanine 5 (Cy5) and fluorescein (FITC or FAM) images are acquired for every field of view. After image acquisition, ImageJ (National Institute of Health; NIH) is used to identify droplets based on their circular boundaries in the bright field images and then measure their fluorescence in the Cy5 and FITC images. The fraction of positive droplets is determined by counting the number with fluorescence signal above a threshold value, divided by the total number of imaged droplets. Samples are tested in triplicate and comprise a minimum of 5000 droplets.

### Encapsulation, PCR, and enrichment of viruses in double emulsion droplets

To make double emulsions, the virus samples are first diluted in phosphate buffered saline. Approximately 1200 T4 virions are mixed with 1.4×10^5^ ФX174 before combining with PCR reagents (Platinum Multiplex PCR Master Mix, Thermo Fisher) and PCR primers (IDT) specific for the species of interest. The middle phase of the double emulsion consists of HFE-7500 fluorinated oil (3M) with 2% (w/w) PEG-PFPE amphiphilic block copolymer surfactant [27], and the carrier aqueous phase of 4% (v/v) Tween 20, 1% (v/v) Pluronic F-68 (Gibco), and 10% (w/v) PEG (molecular weight 35 K) in water [26]. These solutions are loaded into syringes and processed through a microfluidic double emulsion maker using syringe pumps, similar to the single emulsions. The flow rates are 90 μL/h for the virus sample, 80 μL/h for the oil, and 250 μL/h for the outer aqueous phase. The double emulsion droplets exit the device through PE-2 tubing and are collected into a 1.5 mL microcentrifuge tube. Double emulsions produced by this method are ~35 μm in diameter and monodispersed.

To prepare the double emulsion droplets for thermal cycling, the sample is transferred from the 1.5 mL microcentrifuge tube into 0.2 mL PCR tubes, such that each contains 90 μL of emulsion and 10 μL of fresh PCR buffer; the PCR buffer consists of 30 μL of 50 mM MgCl_2_ and 100 μL of 200 mM Tris pH 8.0 and 500 mM KCl and is essential for preventing PCR components from leaching out of the droplets into the carrier phase, in which they are soluble. The sample is cycled on a T100 thermal cycler (Bio-Rad) according to the Platinum Multiplex Master Mix instructions. After thermal cycling, 1× SYBR Green I (Life Technologies) is loaded into the carrier phase, permeating through the double emulsion shell and staining the droplets that have undergone PCR amplification.

A fluorescence-activated cell sorter (FACS) Aria II (BD) is used to sort the emulsions to recover droplets that contain the virus of interest. The FACS chamber temperature is set to 4°C and agitation speed to the highest setting to prevent droplets from sedimenting during the sort. The droplets strongly scatter the FACS laser, requiring a 2× Neutral Density (ND) filter to decrease signal into the detectable range. The microfluidic device produces uniform double emulsions and, consequently, the droplets appear as a compact cluster in forward versus side scatter, making them easy to distinguish from particulate and small oil droplets, which the FACS is instructed to ignore.

The sample is analyzed in batches by diluting 100 μL of emulsion into 200 μL of 2% (v/v) Pluronic F-68 and 1% (w/v) PEG (molecular weight 35 K) in water, and gently mixing using a 200 μL pipette tip. The sample is loaded into the FACS and the double emulsions gated in the Forward Scatter (FSC) and Side Scatter (SSC) channels [22]. To read the SYBR channel relating to amplification, we use a 488 nm laser and a 505LP optical filter (BD Biosciences); the population has two peaks, one with low average intensity representing empty or negative droplets, and another with high average intensity representing SYBR positive droplets, which we gate to recover in either Eppendorf tubes (bulk recovery of target virus from a mixed population) or 96-well plates (recovery of single virion from a mixed sample). We use the strict “purity” setting of the instrument which discards events in which multiple droplets pass through the detection window at the same time.

### Amplification of recovered viral DNA

Sorted droplets are briefly centrifuged to the bottom of the tube. To release nucleic acids, the droplets are ruptured by adding 20 μL of DI water and 50 μL of perfluoro-1-octanol (PFO), and vortexing for 1 min. The sample is centrifuged again, and the aqueous top phase containing the viral DNA removed using a micropipette.

To confirm enrichment of T4 phage in the sorted emulsion, we use quantitative PCR (qPCR). The concentration of viral DNA is too low post-sorting to be reliably detected by bulk qPCR. To address this, we non-specifically amplify the material using digital droplet multiple displacement amplification (ddMDA) prior to qPCR analysis using the Qiagen REPLI-g Single Cell Kit. ddMDA is a non-specific method that amplifies all nucleic acids in a sample uniformly [28]. The sample is incubated with 3 μL of the Denaturation Solution for 10 min at 65°C. After heating, the reaction is halted by adding 3 μL of Stop Solution Mix. 20 μL of the REPLI-g sc Master Mix containing 14.5 μL of REPLI-g sc Reaction Buffer, 4.5 μL of water, and 1 μL of REPLI-g sc Polymerase is added to each sample. The sample is encapsulated into droplets using a 20 μm flow-focus drop maker [29, 30] and HFE-7500 fluorinated oil with 2% (w/w) PEG-PFPE amphiphilic block copolymer surfactant. The emulsion is collected into a 1.5 mL microcentrifuge tube and the reaction incubated at 30°C for 16 h. After incubation, the droplets are ruptured by adding 10 μL of PFO, vortexing, and spinning as above.

### Quantitative PCR analysis of sorted droplets

To confirm that PAVS enriches for bacteriophage T4 over bacteriophage ФX174, we estimate concentrations of both viruses in the sorted and unsorted pools using qPCR (Stratagene Mx3005P, Agilent). The qPCR primer sequences are different from the ones for PAVS detection, so that in-droplet amplification products do not skew qPCR results. Cross-threshold (*C*_*t*_) values for T4 and ФX174 in pre- and post-sorted samples are used to compute an enrichment factor. The amplification reagent for all the qPCR measurements is Maxima SYBR Green Master Mix (Thermo Scientific), and the qPCR primers are listed in Additional file 1: Table S1.

## Results

### PAVS enriches for specific viral species from a heterogeneous sample

**Fig. 1:**
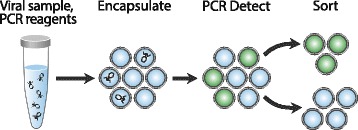
PCR-Activated Virus Sorting (PAVS) workflow. Virus suspensions are encapsulated with PCR reagent and probe in double emulsion droplets, then thermal cycled and stained with SYBR Green. FACS recovers fluorescent droplets containing the viral species of interest, generating an enriched sample that is ready for downstream processing

PCR-Activated Virus Sorting allows specific viruses in a mixed population to be detected and recovered by sorting. This is accomplished by encapsulating the viruses in double emulsion droplets using microfluidic technology and performing PCR in each droplet to probe for sequences of interest. Because the viruses are encapsulated at 0.1 per droplet, most droplets are empty or contain a single virion, in accordance with Poisson statistics (Fig. 1). If the target virus is present in a droplet, PCR amplification occurs, generating a fluorescent signal that can be detected and recovered by FACS. Due to the rapid rate at which microfluidics can encapsulate individual virions in droplets (>1 KHz), millions of single virus particles can be sorted in a few hours.

### Specific detection and quantification of viral genomes

**Fig. 2:**
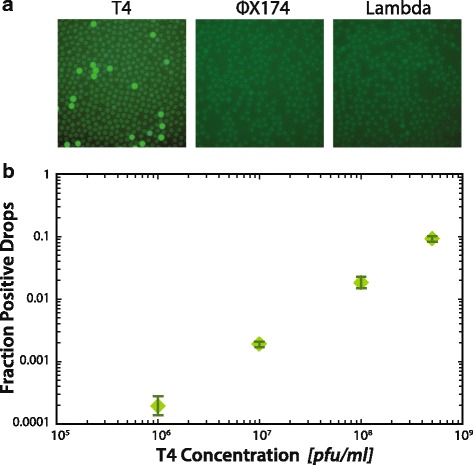
**a** T4, ФX174, and lambda virions are partitioned into droplets with TaqMan primers and probe specific for T4. After thermal cycling, the T4 sample has TaqMan signal while the ФX174 and lambda negative controls have no signal, demonstrating that digital droplet PCR specifically detects target viruses. **b** The fraction of TaqMan positive droplets in digital PCR for T4 is closely related to the input T4 concentration, showing that digital droplet PCR quantitatively measures viral concentration. Error bars indicate the standard deviation for triplicate measurements

To identify a virus in a droplet, PAVS uses a PCR assay interrogating for sequences that exist within the target species. In this way, the PCR primers are analogous to antibodies when sorting cells with FACS, providing a detectable fluorescence signal only when the target species is present. However, whereas generating high affinity antibodies against a target virus can be challenging, especially if it is uncultivable, designing specific PCR primers is straightforward. This makes PAVS general, allowing it to recover any virus of interest to which PCR primers can be designed. To illustrate this, we perform digital TaqMan PCR on samples containing T4, ФX174, and lambda virus, using probes specific for only bacteriophage T4 (Additional file 1: Table S1). The droplets are visualized using epifluorescence imaging after thermal cycling. As expected we observe TaqMan positive droplets in the T4 sample, demonstrating successful amplification when this virus is present (Fig. 2a). By contrast, TaqMan fluorescence is absent in the ФX174 and lambda negative controls (Fig. 2a), confirming that the reaction is specific. This shows that our TaqMan PCR assay can be used to differentiate between single virus particles of these species.

In addition to enabling the detection of specific viruses in a sample, PAVS can count individual virus particles. To demonstrate this, we analyze a dilution series of T4 bacteriophage, reading out the results with fluorescence microscopy and image analysis. We find that, as expected, the fraction of positive droplets is directly proportional to T4 concentration (Fig. 2b). This is due to the viruses being loaded at limiting dilution, such that most droplets are empty but a small fraction contain virus particles. Under such conditions, the viruses are encapsulated individually and the number of droplets containing a virus is approximately equal to the number of viruses in the sample, in accordance with random Poisson encapsulation. As with qPCR, the minimum number of viruses necessary for PAVS depends on the specificity of the TaqMan assay. A strength of TaqMan assays is that they are highly specific, allowing confident detection of rare virus species. For example, in initial tests of this approach, we found that the rate of non-specific amplification in a droplet is 1 in ~100,000, so that viruses less rare than this can be confidently detected. Since we can routinely sort >2 million droplets in a single FACS run, this allows us to detect as few as ~20 virus particles in a sample.

### Multiplexed digital PCR can detect full-length virus genomes

**Fig. 3:**
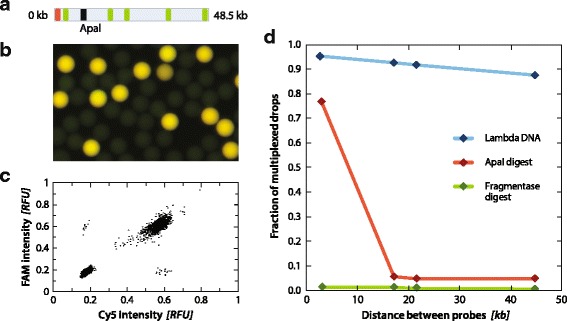
**a** Location of TaqMan PCR Cy5 probe in the lambda genome is shown in *red*, FAM probes are shown in *green*, and the location of the ApaI restriction site is shown in *black*. **b** Representative image of multiplexed PCR emulsion on Lambda DNA. **c** Representative scatterplot of Cy5 and FAM intensities for Lambda DNA. **d** Fraction of multiplexed droplets for Lambda DNA undigested (*blue*), ApaI digested (*red*), and Fragmentase digested (*green*)

A unique and valuable property of PAVS is that it can differentiate between viruses that contain just one target sequence and others that contain multiple. This is possible because TaqMan PCR can be multiplexed using probes targeting different sequences labeled with fluorescent dyes of different color. Hence, viruses containing one sequence will be positive only at one color, whereas those with two sequences will be positive for two colors. These populations can then be separated by gating the fluorescence measurements to recover single- or double-positive droplets. To demonstrate the ability to multiplex the reaction, we synthesize primers and Cy5 TaqMan probes targeting a genomic region near the 5’ end of the lambda genome, and others targeting regions at increasing distances away from the 5’ end. The primer and probe sequences are listed in Additional file 1: Table S1, and a graphical representation of the probe locations with the Cy5 TaqMan probe in red and the FAM TaqMan probes in green is provided in Fig. 3a. Lambda DNA is combined with the PCR reagents and the sample is emulsified using the microfluidic device. After thermal cycling, the droplets are imaged using fluorescence microscopy (Fig. 3b) and analyzed to measure their intensity on the Cy5 and FAM channels (Fig. 3c). The droplets are characterized as positive for both targets (Cy5_+_FAM_+_), positive for one target (FAM + Cy5-, FAM_−_Cy5_+_), or negative for both (FAM_−_Cy5_−_). Each multiplexed PCR is performed in triplicate, containing 5000–8000 droplets.

We observe less multiplexing when probe pairs are far apart, indicating that the probability that two target sequences exist within a given genome decreases for sequences that are more separated (Fig. 3d, blue curve); this implies that the genomes might be partially fragmented. To investigate this further, we perform a negative control in which we digest the genome with a restriction endonuclease cleaving at position 10,086 base pairs (bp), which is between the first and second FAM probes. If fragmentation is the source of lowered multiplexing signal, then the fraction of double-positives should fall precipitously beyond the cleavage point; indeed, this is what we observe, as shown by the red curve in Fig. 3d. As an additional negative control, we digest the lambda genome using a non-specific endonuclease (Fragmentase) producing ~500 bp products, and observe that double-positives are rare for all probe pairs (green curve). This demonstrates that PAVS can characterize the length distributions of viral genomes in a solution and, more generally, the presence of multiple genetic loci in a target virus; this should be useful for studying correlations between loci in single viruses that are on the same linear molecule or on entirely different molecules, such as in segmented virus genomes. PAVS can also be used to characterize the integrity of viral genomes.

### PAVS allows target virus to be sorted out of a mixed population

The PAVS workflow consists of two steps, a first in which target viruses are detected using single virus PCR in droplets, and a second in which the droplets are sorted to recover the target viruses. To demonstrate this, we construct a mixed sample of two bacteriophages, T4 and фX174, at a ratio of 1:999 respectively. This 0.1% T4 spike-in is encapsulated at limiting dilution in droplets with PCR primers specific for T4 phage, thermally cycled, and stained with SYBR Green. If a particular droplet contains T4, the nucleic acids targeted by the PCR primers are amplified and the SYBR stain produces a fluorescent signal that fills the droplet. The fluorescence signal is detected with FACS and the positive droplets sorted into a 1.5 mL microcentrifuge tube.

**Fig. 4:**
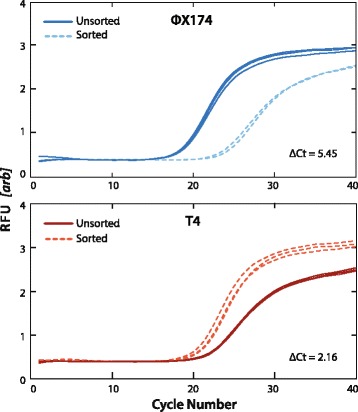
qPCR detection of bacteriophages фX174 and T4 before and after FACS sorting. The shifts in the curves reflect the 2-fold change of the DNA quantity according to the specific primers being tested. Samples tested in triplicate

To validate that the PAVS workflow enriches for T4 over ФX174, we quantify virus concentrations in the sorted and unsorted pools using qPCR. The sorted droplets are ruptured and the viral genomes amplified by ddMDA. Equal concentrations of T4 and ФX174 DNA from the unsorted and sorted emulsions are subjected to qPCR (Fig. 4). The primers used to detect T4 target a different locus than the ones for PAVS sorting (Additional file 1: Table S1). The qPCR curve for T4 shifts to lower cycles post-sorting, demonstrating that T4 has been enriched. By contrast, the curve shifts to higher cycle numbers for ФX174, indicating that this virus has been de-enriched by sorting, as expected. To quantify the degree of enrichment, we compute an enrichment factor *e* defined as,

$$ e=\kern0.5em \frac{\left(n\kern0.5em +\kern0.5em 1\right)\left(\frac{1}{2^{\varDelta {C}_t^{\mathrm{T}4}}}\right)}{\left(\frac{1}{2^{\varDelta {C}_t^{\mathrm{T}4}}}\right)\kern0.5em  + n\left(\frac{1}{2^{\varDelta {C}_t^{\upphi \mathrm{X}174}}}\right)}, $$

where *n* is the ratio of the viral species with respect to one another and Δ*C*_*t*_^T4^ and Δ*C*_*t*_^фX174^ are the differences of cross-threshold values for T4 and фX174, respectively. For this experiment, *n* = 999, Δ*C*_*t*_^T4^ is 2.16, and Δ*C*_*t*_^фX174^ is 5.45, yielding *e* = 9.69, indicating that the final sample is enriched by about tenfold for T4 from an initial concentration of 0.1%. The degree of enrichment is tunable over a large range, as the rarer the target is in the droplets before sorting, the more it is enriched thereafter. Conversely, if the target is abundant, then many droplets will be positive and only a minor enrichment is possible. To increase enrichment, the sample is thus diluted prior to partitioning in droplets, which reduces the rate of co-encapsulation of different viruses and false-positive recovery of off-target species. The enrichment possible is also limited by the false-positive rate of droplet detection, which sets an upper bound to how much the sample can be diluted. The false-positive rate for our TaqMan assay is ~1/100,000 droplets, setting a theoretical upper enrichment limit of ~100,000×; however, the maximum enrichment achieved in practice can also be limited by other considerations, such as a the specificity of assays or the number of positive viruses that must be recovered for downstream characterization.

### PAVS recovery of single virions from a mixed sample

**Fig. 5:**
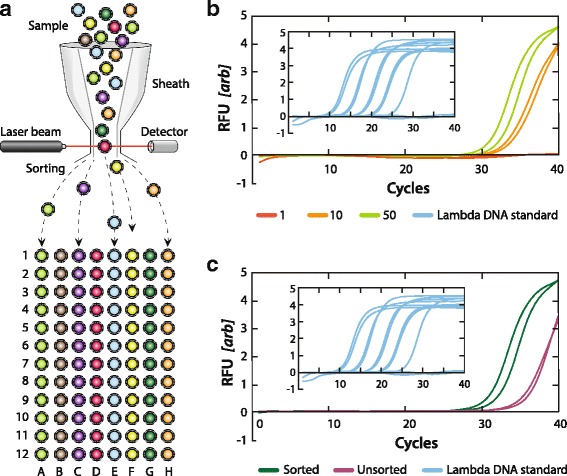
**a** Workflow schematic for targeted virus sorting into well plates using PAVS. **b** qPCR detection of Lambda for 1, 10, or 50 viruses sorted into a well. Positive control of Lambda DNA shown in inset. **c** qPCR curves for 50 sorted or unsorted Lambda viruses per well. Positive control of Lambda DNA shown in inset. Samples tested in duplicate

Common FACS instruments can pool all positive droplets into one well or dispense controlled numbers into different wells, including down to single droplets. This is commonly used to isolate cells for single cell analysis (Fig. 5a). When combined with PAVS, this allows a heterogeneous mixture of viruses to be sorted, to isolate specific virions in the sample, which can then be subjected to additional analyses, such as qPCR. To illustrate this, we sort a sample of lambda virus with PAVS and dispense the positive droplets into wells in controlled numbers (Additional file 1: Table S1, Lambda FWD 2, Lambda REV 2, and Lambda probe 2). We load 1, 10, or 50 positive droplets into each well and analyze the recovered material with qPCR for primers targeting a different portion of the lambda genome than was amplified in the PAVS detection (Additional file 1: Table S1). The *C*_*t*_ values decrease as the number of viruses dispensed increases, indicating that the viruses are present at higher numbers (Fig. 5b). When fewer than 50 viruses are sorted, it is difficult to reliably detect them in the sorted wells; wells with 10 viruses show amplification at *C*_*t*_ values of 33, while single viruses do not amplify above the negative controls.

To confirm that the sorting is specific, we generate qPCR curves for wells containing 50 positive droplets and wells containing 50 unsorted droplets. The qPCR curve shifts left by an average of 4.24 *C*_*t*_ values, demonstrating that the Lambda virus is more abundant in the sorted sample (Fig. 5c). While our results show that single viruses provide too little DNA for detection with standard qPCR, other post-sorting amplification methods could be implemented to improve sensitivity, such as higher efficiency PCR reagents, nested PCR [31], or non-specific ddMDA followed by qPCR [28]. Because DNA can fragment under flow through narrow channels, the ability to perform multiplexed TaqMan assays in the droplets can be used to identify and dispense only intact viral genomes into the wells.

## Discussion and conclusions

PCR-activated virus sorting enables specific detection and isolation of target viruses from a mixed sample, analogous to what is possible with FACS for cells. PAVS has significant advantages over conventional approaches to virus genome study. For example, just as when studying microbes, specific and high throughput virus sorting should be valuable whenever large populations must be analyzed to recover a target species; this requires that only ~ 100 bp of the virus’s genome be known with which to generate identifying TaqMan probes. For a presently-unknown species, this information may be obtained by performing short-read shotgun sequencing on a sample, or consulting existing metagenomic databases.

PAVS can detect viruses residing within host microbes, including bacteria and eukaryotic cells. This is accomplished through the encapsulation of host cells followed by lysis, and subjection to TaqMan PCR for virus detection, which produces a positive signal if the virus is present within the host. The sorted hosts can then be subjected to SSU rRNA profiling or next generation sequencing to identify the species. This capability should be valuable for characterizing virus-host relationships in microbial ecologies or the human microbiome, for example, to determine which viruses infect which hosts – something that is presently extremely challenging due to the inability to culture most viruses and host microbes.

PAVS opens new avenues in the study of virus-mediated disease, such as HIV and EBV, where it enables detection of viral infection within host cells. Unlike oligonucleotide staining, PAVS reliably detects single molecule viral genomes of interest. Moreover, the cells that are positive for infection can be recovered by sorting, allowing their genomes and transcriptomes to be sequenced. For example, the recovered cell lysates can be sequenced to characterize insertion sites of the virus, epigenetic correlations with virus infection, identify genome mutations of the specific virus of interest, or modulation of host cell transcriptome. This will be valuable for studying the basic biology of the virus and to better understand how it survives, replicates, and evades the host immune response.

## Additional file

Additional file 1: Supplemental information. (PDF 3622 kb)

## Abbreviations

bp: base pairs
Cy5: Cyanine 5
ddMDA: digital droplet multiple displacement amplification
*E. coli*: *Escherichia coli*
EBV: Epstein-Barr Virus
FACS: fluorescence-activated cell sorter
FITC/FAM: fluorescein
FSC: Forward Scatter
HIV: Human Immunodeficiency Virus
IDT: Integrated DNA Technologies
ND: neutral density
NEB: New England Biolabs
NIH: National Institute of Health
PAVS: PCR-Activated Virus Sorting
PCR: Polymerase chain reaction
PDMS: Poly(dimethylsiloxane)
PEG-PFPE: Polyethylene glycol-perfluoropolyether
PFO: perfluoro-1-octanol
qPCR: quantitative PCR
rRNA: ribosomal RNA
SSC: Side Scatter
