# Supplementary material for: Peering below the diffraction limit: robust and specific sorting of viruses with flow cytometry
Source: Virol J. 2016 Dec 1;13:201. doi: 10.1186/s12985-016-0655-7 (PMC5131442; doi:10.1186/s12985-016-0655-7)
Supplement: Additional file 1: — Supplemental information. (PDF 3622 kb) [file 12985_2016_655_MOESM1_ESM.pdf]

Probe	Sequence
T4 probe	5'-/56-FAM/TCGCATTCT/ZEN/TCCTCTGATGGAGCA /3IABkFQ/ -3'
T4 FWD	5’-CCACAACTAACCGAGGAAGTAA-3’
T4 REV	5’-TGCGATATGCTATGGGTCTTG-3’
Lambda probe 1	5’- /5Cy5/TCTGCCCGTGTCGGTTATTCCAAA/3IAbRQSp/-3’
Lambda FWD 1	5’- GACTACATCCGTGAGGTGAATG-3’
Lambda REV 1	5’- TTGCGCTGCTTATGCTCTAT-3’
Lambda probe 2	5’- /56-FAM/TGGGTGTCTCGTATGAGCAGCTTT/3IABkFQ/-3’
Lambda FWD 2	5’- TAACGGCTACTCCGTGTTTG-3’
Lambda REV 2	5’- AGCTCATCTGGGCGTAATTC-3’
Lambda probe 3	5’- /56-FAM/TGAGCAGAC/ZEN/CAAAGACGGCAAACA/3IABkFQ/-3’
Lambda FWD 3	5’- TGGAAGGATGCCAGTGATAAG-3’
Lambda REV 3	5’- TCCATGCTGAGGCCAATA C-3’
Lambda probe 4	5’- /56-FAM/CTCGTTGCT/ZEN/GGAAGCCTGGAAGA/3IABkFQ/-3’
Lambda FWD 4	5’- CAGGTAGCCAGTGAGCATATT-3’
Lambda REV 4	5’- GTTCAGCAACACCCGATACT-3’
Lambda probe 5	5’- /56-FAM/ATACTGAGC/ZEN/ACATCAGCAGGACGC/3IABkFQ/-3’
Lambda FWD 5	5’- GCCCTTCTTCAGGGCTTAAT-3’
Lambda REV 5	5’- CTCTGGCGGTGTTGACATAA-3’
Lambda probe 6	5’- /56-FAM/TATCCGTCA/ZEN/GGCAATCGACCGTTG/3IABkFQ/-3’
Lambda FWD 6	5’- GTGGCATTGCAGCAGATTAAG-3’
Lambda REV 6	5’- GGCAGTGAAGCCCAGATATT-3’
**Primers for qPCR**	**Sequence**
T4 FWD	5’-ACCCGGACCAAAATCTCGAC-3’
T4 REV	5’-GCGCAGTAGTCCGTGAATTG-3’
ΦX174 FWD	5’-TTCTGTGCCGCGTTTCTTTG-3’
ΦX174 REV	5’-AAACAGGGTCGCCAGCAATA-3’
Lambda FWD	5’-CGTGATGGAGCAGATGAAGAT-3’
Lambda REV	5’-GTATCCAGCTCACTCTCAATGG-3’

Key:
/56-FAM/ - isomer derivative of fluorescein attachment for oligonucleotides
/5Cy5/ - Cy5 fluorescent dye attachment for oligonucleotides
/ZEN/ - internal quencher for TaqMan and qPCR probes
/3IABkFQ/ - Iowa Black quencher ideal for use with fluorescein
/3IAbRQSp/ - Iowa Black quencher ideal for use with Cy5

Supplemental Table 1

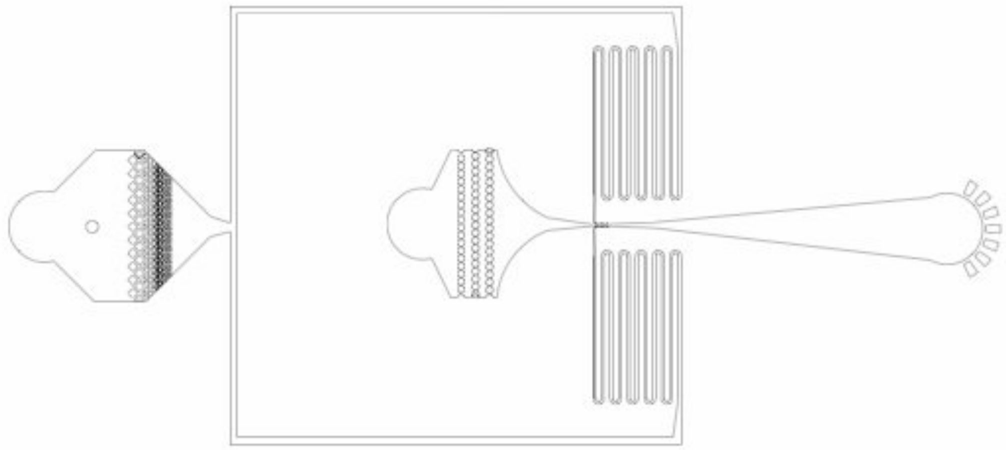

Supplemental Figure 1 – Diagram of single emulsion dropmaker.

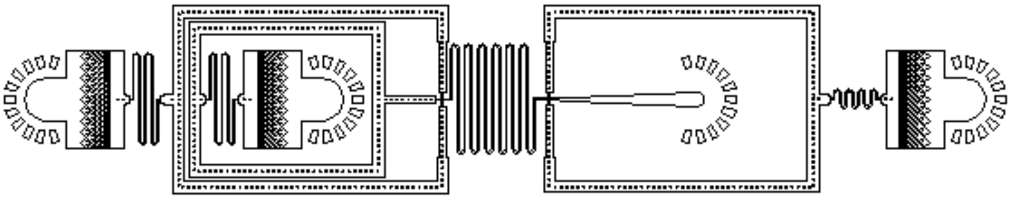

Supplemental Figure 2 – Diagram of double emulsion dropmaker.

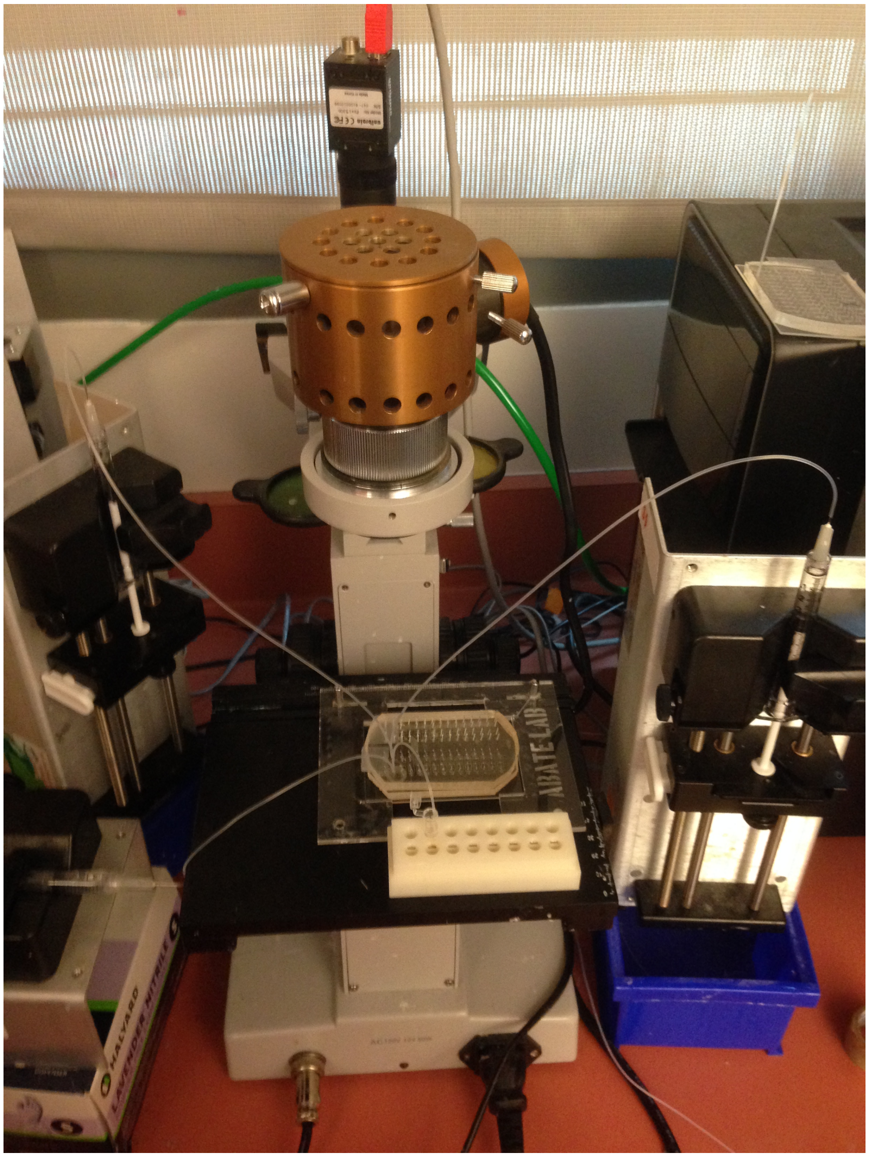

Supplemental Figure 3 – Image of dropstation.

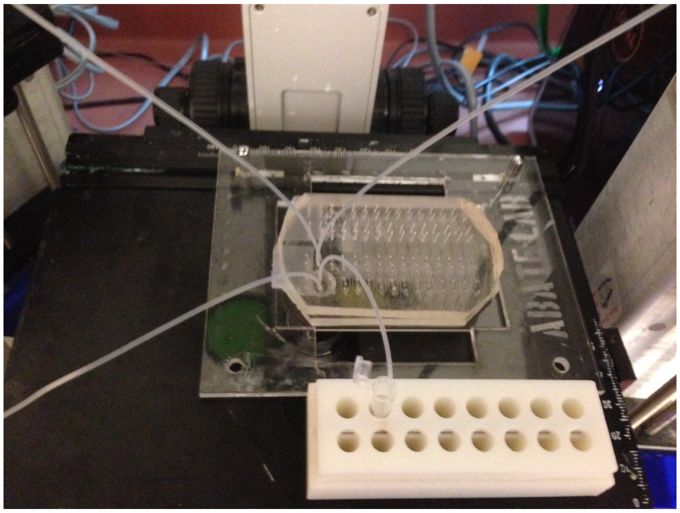

Supplemental Figure 4 – Close-up image of dropstation.
